# On the Use of Carbonate of Iron in Tic Douloureux

**Published:** 1823-02

**Authors:** Stewart Crawford

**Affiliations:** Bath.


ART. III.—*On the Use of Carbonate of Iron in Tic Douloureux.* By Dr. STEWART CRAWFORD, of Bath. Communicated by BENJAMIN HUTCHINSON, Esq.

*The Circus, Bath; Oct.* 1822.

MY DEAR SIR,

I OWE you many apologies for having delayed so long an answer to your polite letter; but the truth is, that my notes of the case respecting which you wish to be informed were mislaid, and it was not until yesterday that I was able to find them. The detail which follows of the symptoms and treatment of the lady who was my patient, suffering from the tic douloureux, is much at your service; and the efficacy of the carbonate of iron, which you have so happily introduced, has been so strongly marked, that I think you will consider it a case which merits your attention.

Mrs. P. first experienced an attack of the tic douloureux in her sixty-ninth year. The left side of the face was the one affected. At the commencement of the disorder, the paroxysms were slight and unfrequent; but after some time their severity was much aggravated, and the intervals of ease were fewer. I have seen several instances of this painful malady, but none in which the sufferings were greater. I cannot convey a more faithful account of what my patient endured, than by transcribing her own account, taken from a journal kept by her from the commencement of her complaint.

"At first the spasm felt as if a red-hot knitting needle ran through my nose and eyes to the top of the head,—as if my brain was on fire. I could not bear any thing to touch that side of my face; and even a hair touch would rouse the spasm. My nose and my eye streamed with water, and I even feared to wipe it away. For two nights I had difficulty to get my night-cap on; nor, during this long period of torture, could I blow my nose, but kept a handkerchief to pat it gently."

On being desired to see this lady, I directed the arsenical solution to be taken in doses of six drops three times a-day, largely diluted with barley-water. After persevering for some time in this plan of treatment, the disease was completely subdued, and my patient remained free from it for two years. At the end of this time a slight attack took place, which speedily yielded to the arsenical solution.

For six years Mrs. P. escaped without any attack of her former disease; but, on the commencement of the seventh, it again annoyed her. Confiding in the efficacy and safety of the medicine which had hitherto relieved her, she took it as formerly, without consulting me. Unfortunately, it now produced no alleviation of her sufferings, but disordered her stomach exceedingly. My assistance was now desired; and, wishing to try the solution again, I directed her to take it after a few days' interval; but, from its deleterious effects on the stomach, I was soon obliged to desert it, although small doses were given. Under these circumstances, having lately read your publication, recommending the carbonate of iron in this disease, I prescribed it for my patient. I began with doses of a scruple three times a-day, and gradually increased it to one drachm each time of taking it. In three weeks from the commencement of this treatment, the complaint had entirely ceased: indeed, shortly after it was begun, the severity of the pains, and their frequency of recurrence, were much lessened. To evince more strongly the power which the carbonate of iron possessed over the complaint, it may not be improper to mention that, in consequence of a mistake in the person who dispensed the medicine, carbonate of potass was substituted, for a few days, instead of carbonate of iron: during this time the spasms returned with their usual violence and frequency; but, on the mistake being discovered and rectified, the same good effects followed as formerly. Mrs. P. continued free from any attacks for nearly six months: since then she has experienced several slight returns of the disorder, but they are speedily subdued by the carbonate of iron, which she takes, without any inconvenience, in doses of one drachm three times a-day.

As I conceive every well-authenticated case of the beneficial effects of the medicine you have recommended, must be interesting to you and the medical profession, I would mention that, lately, in conversation with my much-respected friend, Dr. Davis, of this city, on the disease in question, he stated to me his success in the case of a lady, his patient, for whom he prescribed the carbonate of iron, at my request. Dr. Davis has been so good as to transcribe from his journal his notes of the case, which are as follows:

"My patient, Mrs. H—, æt. 65, has the usual symptoms of tic douloureux on the right side of the face. A careful examination of the teeth caused no suspicion of the pain being occasioned by caries within the mouth. She was ordered ten grains of Dover's powder every night at bed-time, and extract of hemlock during the day. This plan commenced on the 19th of May, 1820, and was continued until the 20th of the following month; when, being no better, she began to take two scruples of the carbonate of iron, with five grains of the compound cinnamon powder, morning and noon. At the end of a fortnight, I had the pleasure to hear that she was relieved. She was advised to continue the use of the remedy a week longer; which, I believe, she did. Having seen her about two months ago, I am enabled to add, that she has enjoyed good health ever since she left off taking the powders."

Sincerely wishing that more ample experience may completely establish the efficacy of the medicine you have proposed for the relief of this hitherto intractable disease, I have the honour to be, Sir, your very obedient servant,

STEWART CRAWFORD, M.D.

*To B. Hutchinson, Esq. Southwell.*

